# Establishment of Normative Retinal Nerve Fiber Layer Thickness in Healthy Koreans Using Huvitz Optical Coherence Tomography and Comparison with Cirrus OCT

**DOI:** 10.3390/jcm14124258

**Published:** 2025-06-15

**Authors:** Heesuk Kim, Ji Eun Park, Wungrak Choi

**Affiliations:** 1 Institute of Vision Research, Department of Ophthalmology, Yonsei University College of Medicine, Seoul 03722, Republic of Korea; kimhseye@yuhs.ac; 2 Yonsei University College of Medicine, Seoul 03722, Republic of Korea; jieun.park19@med.yuhs.ac

**Keywords:** retinal nerve fiber layer thickness, optical coherence tomography, device-specific normative data, ethnicity-specific normative data, glaucoma diagosis, age-related retinal nerve fiber layer thinning

## Abstract

**Objectives:** The purpose of this study was to evaluate the diagnostic accuracy of glaucoma by establishing normative data on retinal nerve fiber layer (RNFL) thickness, specifically for healthy Koreans, using Huvitz spectral-domain optical coherence tomography (OCT). This study also aimed to compare the obtained RNFL thickness data with normative values provided by the Cirrus OCT system to identify any device-specific differences that could impact glaucoma diagnosis. **Methods**: This prospective observational study included 148 healthy participants aged 20–69 years at Gangnam Severance Hospital. Participants underwent comprehensive ophthalmologic evaluations, including RNFL thickness measurements using Huvitz OCT, which were compared with existing normative Cirrus OCT data. RNFL thickness was analyzed by quadrant (superior, inferior, nasal, and temporal) and clock-hour sectors. Statistical analysis included one-way analysis of variance (ANOVA) for group comparisons and linear regression to assess age-related changes. **Results**: The average RNFL thickness was 91.13 ± 13 μm, with the thickest measurements in the superior quadrant (111.85 ± 18.53 μm) and the thinnest in the nasal quadrant (68.35 ± 20.03 μm). Significant age-related thinning was observed across all quadrants, particularly the superior and inferior quadrants. Comparison with the Cirrus OCT system revealed significant differences, with the Huvitz OCT results showing thinner RNFL in the superior and inferior quadrants. **Conclusions**: This study established normative RNFL thickness data in healthy Koreans using Huvitz OCT, providing essential reference data for clinical glaucoma diagnosis. The differences between Huvitz and Cirrus OCT systems underscore the need for device- and population-specific normative data to improve diagnostic accuracy in glaucoma management.

## 1. Introduction

Glaucoma is a leading cause of irreversible blindness characterized by retinal ganglion cell degeneration resulting in structural changes in the retinal nerve fiber layer (RNFL) and optic nerve head. By 2040, 11.8 million people worldwide are expected to be affected, largely owing to aging [1,2]. Early detection is critical, and advances in imaging technology have revolutionized glaucoma management [3].

Optical coherence tomography (OCT) is indispensable in diagnosing and monitoring glaucoma as well as a variety of retinal diseases [4,5]. OCT is noninvasive and provides high-resolution cross-sectional images of the retina [6,7]. Spectral-domain OCT (SD-OCT) has enhanced speed and resolution, enabling detailed retinal structure analysis; it measures RNFL, ganglion cell complex (GCC), and inner plexiform layer (IPL) thicknesses, which are key indicators of glaucomatous damage [8,9,10]. In glaucomatous eyes, these thicknesses are significantly reduced, and the RNFL thickness in each quadrant is useful for detecting glaucomatous degeneration [11].

Evaluating normative data remains challenging owing to structural overlap between healthy and early glaucomatous eyes; measurements may vary by ethnicity, sex, or age [8,12,13,14]. Age is a critical factor in RNFL evaluation, showing RNFL thinning with age [15,16,17,18,19,20,21,22,23,24,25,26,27,28,29,30]. While OCT devices like the Cirrus OCT (Carl Zeiss Meditec Inc., Jena, Germany) provide age-matched color probability maps, they often overlook ethnic variations, potentially leading to inaccurate clinical assessments.

Recently, a new swept-source OCT device (HOCT-1F, Huvitz Co., Ltd., Anyang, Korea) has become available. As normative RNFL thickness values may vary across different OCT devices due to technical and methodological differences, it is important to establish normative RNFL data specifically measured by this device. In this study, we aimed to measure normative RNFL thickness data in healthy Korean individuals using the Huvitz OCT device and compare these findings with normative data obtained from the widely used Cirrus OCT device. Through this comparative analysis, we sought to identify potential ethnic and device-specific differences, thereby enhancing the clinical utility and interpretation of RNFL measurements in glaucoma diagnosis and management.

## 2. Materials and Methods

### 2.1. Study Design and Participants

This prospective observational study was conducted at Gangnam Severance Hospital between November 2023 and February 2024, following the principles of the Declaration of Helsinki. The study was approved by the Gangnam Severance institutional review board (number: 3-2023-0228), and written informed consent was obtained from all participants. The study included 148 healthy participants (76/72 male to female ratio), aged 20–69. Eligibility required intraocular pressure below 21 mmHg, spherical refractive error within ±6.0 D, cylindrical astigmatism within ±3.0 D, and best corrected visual acuity (BCVA) of at least 20/20 (participants <60 years) or 20/25 (participants 61–69 years). Exclusion criteria were significant ocular pathologies, including severe myopic degeneration, glaucoma, or optic nerve abnormalities, as well as prior corneal refractive surgery, ophthalmic surgery including cataract extraction, central refractive media opacity, pregnancy, or breastfeeding.

A detailed history, including medical history and family history of glaucoma, was thoroughly assessed. Additionally, optic disc photographs of all participants were independently reviewed by two glaucoma specialists, and only those participants whose optic nerve head was unanimously classified as normal were included in the analysis.

Each eye of every participant was assessed separately to determine eligibility, and any eligible eye was included in the final analysis.

### 2.2. Ophthalmic Examination

All participants underwent comprehensive ophthalmic evaluations, including BCVA, spherical and cylindrical errors, slit-lamp examination, and non-mydriatic fundus examination, before undergoing OCT scanning. Intraocular pressure was measured using a noncontact tonometer (NCT; KT-800 tonometer; Kowa, Tokyo, Japan).

### 2.3. Optical Coherence Tomography

The Huvitz OCT(HOCT-1F) is a swept-source OCT with a speed of 60,000 A-scans/s, while the Cirrus OCT, used for comparison in this study, is a spectral-domain OCT with a faster scanning speed of 100,000 A-scans/s. All participants underwent a single wide scan (12 mm × 9 mm) centered on the fovea in both eyes using the Huvitz OCT, which covered the macula and peripapillary RNFL regions. The Macular Wide scan protocol of the Huvitz OCT includes multiple resolutions (A-scan × B-scan): 1024 × 64, 512 × 128, 512 × 96, 512 × 64, 256 × 256, and 256 × 128 pixels, and scans can be obtained in both horizontal and vertical directions. The scan was analyzed using automated segmentation algorithms to assess retinal layer thicknesses, including the GCC and IPL. Measured parameters included peripapillary RNFL thickness (360° TSNIT), cup-to-disc ratio, macular layer, macular retinal pigment epithelium (RPE), GCC, and IPL thickness. Detailed 3D macular analyses, including thickness maps and charts, were automatically generated. For optic disc analysis, the Huvitz OCT defines the disc area based on RPE endpoints rather than Bruch’s membrane opening. Two RPE endpoints were connected to define the disc area. Subsequently, the cup area was defined by the intersection of the internal limiting membrane (ILM) and a reference plane located 150 µm above this connecting plane. The rim area was calculated by subtracting the cup area from the disc area. Cup depth was measured as the vertical distance from the ILM to the reference plane at the cup border. All disc parameters were automatically measured by the device software. Segmentation errors in disc and cup boundaries were manually corrected when identified. Scans with a signal strength index (SSI) below 7 or data loss exceeding 10% of the scan area were excluded as recommended in the user manual [31].

### 2.4. Statistical Analysis

Descriptive statistics were calculated, including mean, standard deviation, and range. RNFL thickness was stratified by age group (20–29, 30–39, 40–49, 50–59, and 60–69 years). One-way analysis of variance (ANOVA) was used to analyze differences across age groups, followed by Tukey’s test for multiple comparisons. Pearson’s correlation was used to evaluate age and RNFL thickness. A linear regression model assessed age effects on RNFL thickness, with Z-scores used to calculate predicted thickness ranges: <1% (Z-score < −2.327), 1–5% (−2.327 ≤ Z-score < −1.645), 5–95% (−1.645 ≤ Z-score ≤ +1.645), and 95–100% (Z-score > +1.645). Macular thickness and optic nerve head parameters were also analyzed by sector using descriptive statistics, correlation analysis with age, and percentile ranges.

To compare RNFL thickness measurements between the Huvitz OCT and the Cirrus OCT, weighted averages and standard deviations were calculated for each device. Age-stratified comparisons between the two devices were performed using paired *t*-tests, and *p*-values < 0.05 were considered statistically significant. All analyses were performed using R software (version 4.3.1; R Core Team, Vienna, Austria).

## 3. Results

### 3.1. Baseline Characteristics and Population Distribution

This study included 262 eyes from 148 individuals; 34 eyes were excluded owing to low SSI, >10% data loss, or history of ocular abnormalities (Figure S1). The mean age was 43.65 years [SD: 12.89 years], and the sex distribution was balanced. The mean disc area was 1.95 mm^2^ [SD: 0.53 mm^2^] (range: 0.38–4.07 mm^2^), the mean rim area was 0.89 mm^2^ [SD: 0.32 mm^2^] (range: 0.21–0.65 mm^2^), the mean cup volume was 0.26 mm^2^ [SD: 0.22 mm^2^] (range: 0.01–0.04 mm^3^), and the mean disc volume was 0.11 mm^3^ [SD: 0.53 mm^3^] (range: 0.04–0.2 mm^3^). The age distribution was as follows: 20s (19.59%), 30s (19.59%), 40s (27.03%), 50s (20.95%), and 60s (12.84%); using a chi-squared test to compare this with official population distribution percentages by the National Statistical Office [32] showed no statistically significant differences (*p* = 0.904; Table 1).

### 3.2. RNFL Thickness Measurements

The average RNFL thickness was 91.13 μm [SD: 13 μm] (range: 44.59–127.13 μm), with the highest thickness in the superior quadrant (111.85 μm [SD: 18.53 μm]), followed by the inferior (110.40 μm [SD: 19.90 μm]), temporal (73.93 μm [SD: 10.89 μm]), and nasal quadrants (68.35 μm [SD: 20.03 μm]; Table 2).

ANOVA revealed significant differences in RNFL thickness across age groups (*p* < 0.01), with the highest thickness in the 40–49 age group (95.69 μm [SD: 10.32 μm]) and the thinnest in the 60–69 age group (80.07 μm [SD:14.32 μm]). Tukey’s test revealed significant reductions in RNFL thickness in the 60–69 age group compared to other groups (Supplementary Table S1). The 50–59 and 60–69 age groups also showed significant differences compared with the 40–49 group. Quadrant analysis revealed a significant reduction in RNFL thickness in the 60–69 group compared to those in age groups under 40 years.

### 3.3. Analysis by Clock-Hour Sectors

**Figure 1:**
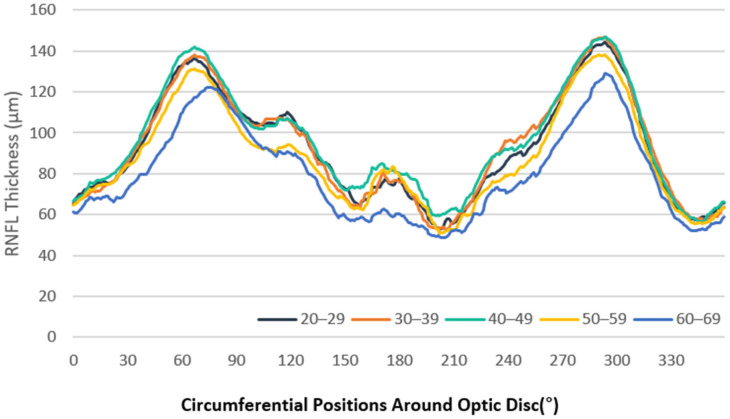
Age-stratified RNFL thickness map (μm) using the TSNIT method.

**Figure 2:**
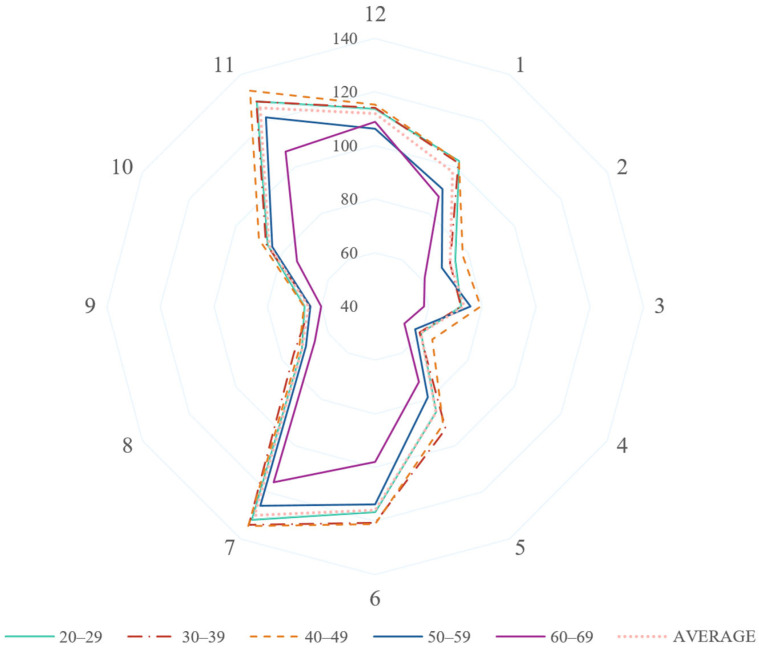
Age-stratified RNFL thickness (μm) map by clock-hour directions.

Clock-hour sector analysis indicated the highest average RNFL thickness in the 7 o’clock sector (129.82 μm [SD: 21.35 μm]) and the lowest in the 4 o’clock sector (59.66 μm [SD: 19.12 μm]; Table 3). Age-related thinning was prominent in sectors 6, 7, and 11, with general thinning observed in older individuals (Figure 1 and Figure 2).

### 3.4. Correlation Analysis

A significant negative correlation was seen between age and RNFL thickness (r = −0.27, *p* < 0.001), particularly in the inferior (r = −0.26, *p* < 0.001) and superior (r =−0.23, *p* < 0.001) quadrants. The nasal quadrant showed a weak, nonsignificant correlation (r = −0.12, *p* = 0.061). The strongest negative correlations were observed in clock-hour sectors 1, 5, 6, 7, 10, and 11 (all *p* < 0.01; Table 4). Percentile analysis results are presented in Supplementary Tables S2–S4.

### 3.5. Macular Thickness Measurement

Macular thickness was measured using the Early Treatment Diabetic Retinopathy Study (ETDRS) grid. The thickest macular layer was seen in the inner nasal sector (321.76 μm [SD: 15.03 μm]) and the thinnest in the center sector (243.7 μm [SD: 19.25 μm]; Table 5). The average GCC thickness was 111.70 ± 7.47 μm, with the highest thickness in the superior-nasal sector (118.61 μm [SD: 8.46 μm]) and lowest in the temporal-superior sector (100.52 μm [SD: 7.62 μm]; Table 6). IPL thickness averaged 111.79 μm [SD: 7.62 μm], with no significant difference between superior and inferior sectors. The average RPE thickness was 281.77 μm [SD: 11.91], with the highest thickness in the superior sector (283.83 μm [SD: 12.32 μm]) and the lowest in the fovea sector (196.32 μm [SD: 17.30 μm]).

Percentile analysis results are provided in Supplementary Tables S5 and S6. Linear regression revealed no significant negative correlations between age and overall macular thickness. However, significant negative correlations were observed between GCC thickness and age in the superior-nasal (r = −0.14, *p* = 0.027) and nasal-inferior sectors (r = −0.16, *p* = 0.011; Table S7).

### 3.6. Comparison of RNFL Thickness Measurements

Because we did not have access to the raw normative database of the Cirrus HD-OCT for direct analysis, we compared our results with normative values reported in previously published studies based on Korean populations using the Cirrus HD-OCT [16,17] (Table 7). While the Cirrus OCT provides age-matched probability maps, these reported normative values do not explicitly account for ethnic differences, which may influence cross-population comparisons. Although the three groups had similar age ranges, the age distribution differed significantly. Cirrus OCT 1 normative data [17] had a higher proportion of participants in the 30–39 (28.14%) and 50–59 age groups (25.42%) than the Huvitz OCT (19.47% and 20.61%) and Cirrus OCT 2 data [16] (20.53% and 18.54%). The Huvitz data included more participants in the 40–49 years age group (29.01%) than both the Cirrus OCT 1 (30.51%) and Cirrus OCT 2 data (20.53%).

The Huvitz OCT showed the lowest mean RNFL thickness (91.13 μm [SD: 13.00 μm], followed by Cirrus OCT 2 (95.08 μm [SD: 3.47 μm]) and Cirrus OCT 1 (98.26 μm [SD: 9.27 μm]). Using age-standardized weighted averages, adjusted *p*-values confirmed that these differences were statistically significant in the superior and inferior quadrants (all *p* < 0.001), with no significant differences in the nasal quadrant (*p* > 0.05).

Further analysis showed consistent significant differences for the superior and inferior quadrants between devices for all age groups (*p* < 0.001), with no significant differences between devices in the nasal quadrant across any age group (*p* > 0.05). In the temporal quadrant, significant differences were observed between the Huvitz OCT and both the Cirrus OCT 1 and 2 in participants 40 years and older (*p* < 0.05), while no significant differences were detected in participants younger than 40 years (Supplementary Table S8).

## 4. Discussion

This study established normative data for RNFL thickness in healthy Korean participants using the Huvitz OCT and compared these findings with previously reported normative data obtained using the Cirrus OCT in Korean populations. RNFL thickness can vary across ethnicities, making population-specific normative data crucial for accurate glaucoma diagnosis and management. Our RNFL thickness measurements followed an expected pattern in this population, and these normative data will serve as an important reference for clinicians treating Korean patients.

We observed age-related RNFL thinning at an average rate of −2.7 μm per decade, consistent with previous studies reporting rates from −1.6 to −3.65 μm per decade [12,13,14,15,16,18,19,20,21,22,23,24,25,26,27,28]. This highlights the importance of age-adjusted normative data to distinguish normal age-related changes from glaucomatous damage. Interestingly, we noted that the 40–49 age group exhibited the greatest average RNFL thickness. Although RNFL generally thins progressively with age, this unexpected finding may reflect selection bias or cohort effects, whereby individuals within this age group possibly had better ocular health than other groups included. Alternatively, biological variations or subtle methodological variations may have contributed to this observation. Further longitudinal studies are necessary to investigate and confirm this phenomenon.

Regional differences in RNFL thinning could be attributed to inherent structural and functional characteristics of retinal nerve fibers. A previous study has suggested that age-related axonal loss predominantly affects smaller nerve fibers, which may contribute differently to thinning across retinal regions [33]. Although retinal ganglion cell axons in the inferior and nasal quadrants are generally thicker than those in the superior or temporal regions [34], the inferior quadrant paradoxically shows the most prominent thinning with aging. This unexpected pattern may be related to the inferior quadrant’s higher metabolic demand and resultant susceptibility to oxidative stress, vascular supply differences, or potential vulnerability associated with early glaucomatous damage, even in clinically healthy eyes. Additionally, the relatively greater baseline thickness in the inferior quadrant could contribute to larger measurable absolute reductions over time. Further research is necessary to elucidate these mechanisms clearly. In contrast, the nasal quadrant typically exhibits the least amount of age-related RNFL thinning. One possible explanation is its limited functional contribution to central vision, possibly reducing metabolic demands and structural vulnerability over time. A previous study attributed aging differences in average RNFL between both eyes to the dominant eye consuming more oxygen, owing to its higher workload [20]. Similarly, reduced metabolic demand in the nasal quadrant may contribute to its relative resistance to age-related changes. Moreover, the lower baseline thickness and measurement limitations in this region could further obscure subtle changes.

Previous cross-sectional studies support these observations. Celebi and Miraza [15] reported an average RNFL thickness change of −3.65 μm per decade using Cirrus OCT, with the fastest thinning occurring in the inferior quadrant (−5.75 μm per decade) and the slowest in the nasal quadrant (−1.41 μm per decade). This aligns with findings from another Cirrus OCT study [16], supporting consistent trends across studies. Comparable quadrant-specific findings have been reported using Stratus OCT [18,19]. However, there remain notable differences in absolute RNFL thickness measurements and rates of thinning across studies. These discrepancies likely arise from variations in ethnic backgrounds of study populations, study designs, and measurement methodologies. This underscores the importance of developing normative databases specific to ethnicity and to the particular OCT device used, ensuring more accurate and reliable clinical interpretations.

We also compared our findings with previously published normative data using Cirrus OCT in healthy Korean populations [16,17] to evaluate consistency and reliability. Despite inherent test–retest variability of OCT measurements (typically up to up to 5 µm [35]), significant differences were observed between devices, especially in the superior and inferior quadrants, with the Cirrus OCT consistently measuring thicker RNFL values than the Huvitz OCT. These differences persisted even after adjusting for discrepancies in age distribution among study populations, suggesting that methodological and technical factors contributed significantly to the observed differences. For instance, Cirrus OCT utilizes separate macular and optic disc scans, whereas the Huvitz OCT simultaneously acquires both regions using a single wide scan. Previous studies have suggested that separate analyses of peripapillary RNFL and macular regions may offer increased accuracy, particularly with swept-source OCT devices [36]. Thus, differences in scanning protocol and segmentation algorithms are likely responsible for the variations noted in RNFL measurements across different OCT devices. Clinicians should therefore exercise caution when interpreting RNFL thickness measurements obtained from different OCT devices, even within similar populations.

Several limitations should be acknowledged. First, axial length measurements and visual field assessments were not conducted, both of which could potentially influence RNFL measurements. However, we excluded participants with a history of cataract surgery and applied strict refractive criteria to minimize biometric variability. Furthermore, thorough optic nerve evaluations were performed by two glaucoma specialists to rigorously exclude any participants with suspected glaucomatous changes. In addition, the relatively small sample size, particularly in older age groups, may limit the generalizability of our findings. Future research should incorporate axial length measurements, visual field tests, simultaneous multi-device comparisons, and longitudinal analyses to further refine and validate these normative data.

## 5. Conclusions

Our study provides normative peripapillary RNFL and multiple retinal macular thickness data for healthy Korean individuals using the Huvitz OCT device. Comparisons with existing Cirrus OCT normative data revealed significant device-specific differences, underscoring the necessity for normative databases tailored to specific ethnic populations and OCT technologies. These normative data will enhance the clinical accuracy of glaucoma diagnosis and management, emphasizing the importance of ethnicity-specific and device-specific reference standards.

## Figures and Tables

**Table 1:** Baseline characteristics and population distribution.

Baseline Characteristics											
**Parameters**											
Number of eyes					262						
Age, mean (SD), range					43.65 (12.89), 20–69						
Sex (M/F)					135/127						
Laterality (R/L)					133/129						
Disc Area (mm^2^), mean (SD)					1.95 (0.53)						
Rim area (mm^2^), mean (SD)					0.89 (0.32)						
Cup volume (mm^3^), mean (SD)					0.26 (0.22)						
Disc volume (mm^3^), mean (SD)					0.11 (0.53)						
**Population Distribution**											
**Age Group (*N*)**	**20s (29)**		**30s (29)**		**40s (40)**		**50s (31)**		**60s (19)**		***p*** **-Value**
**Distribution Percentage (%)**	**19.59%** **(18% ^§^)**		**19.59%** **(21% ^§^)**		**27.03%** **(24% ^§^)**		**20.95%** **(23% ^§^)**		**12.84%** **(15% ^§^)**		
Sex	F	M	F	M	F	M	F	M	F	M	0.904
Subjects (*N*)	15	14	13	16	20	20	13	18	11	8	
Eyes (*N*)	24	25	24	27	37	39	23	31	19	13	

^§^ Population distribution percentages provided by the National Statistical Office. SD: standard deviation.

**Table 2:** RNFL thickness by quadrant.

Age Group	RNFL Thickness (μm)				
	Average	Superior	Nasal	Inferior	Temporal
All, mean (SD)	91.13 (13.00)	111.85 (18.53)	68.35 (20.03)	110.40 (19.90)	73.93 (10.89)
20–29, mean (SD)	92.35 (10.24)	114.74 (15.27)	68.74 (20.62)	111.30 (17.26)	74.62 (11.14)
30–39, mean (SD)	93.39 (15.50)	114.68 (22.63)	67.75 (21.14)	115.75 (25.45)	75.36 (9.10)
40–49, mean (SD)	95.69 (10.32)	116.87 (13.20)	73.89 (20.85)	115.48 (16.33)	76.51 (10.31)
50–59, mean (SD)	88.04 (10.89)	105.98 (15.93)	67.22 (17.65)	106.24 (17.06)	72.73 (10.24)
60–69, mean (SD)	80.07 (14.32)	100.91 (24.04)	57.44 (14.60)	95.46 (17.72)	66.47 (12.50)

RNFL: retinal nerve fiber layer, SD: standard deviation.

**Table 3:** RNFL thickness by clock-hour sectors.

Age Group	Clock Hour Sector	RNFL Thickness (μm), Mean (SD)	Clock Hour Sector	RNFL Thickness (μm), Mean (SD)
All	12	112.02 (25.07)	6	115.88 (27.48)
	1	98.00 (23.82)	7	129.82 (21.35)
	2	72.20 (23.62)	8	71.50 (13.27)
	3	73.18 (28.48)	9	64.75 (10.05)
	4	59.66 (19.12)	10	85.53 (16.05)
	5	85.51 (22.76)	11	125.54 (22.67)
20–29	12	113.54 (22.86)	6	116.65 (24.83)
	1	102.54 (23.36)	7	131.85 (20.95)
	2	74.56 (22.24)	8	71.45 (11.52)
	3	71.91 (28.46)	9	66.30 (10.00)
	4	59.74 (20.96)	10	86.11 (16.81)
	5	85.40 (20.46)	11	128.13 (19.35)
30–39	12	113.95 (30.98)	6	120.68 (34.15)
	1	101.86 (28.66)	7	133.89 (24.28)
	2	72.13 (26.15)	8	75.01 (12.99)
	3	71.98 (29.51)	9	64.15 (8.51)
	4	59.15 (20.67)	10	86.92 (12.33)
	5	92.66 (29.68)	11	128.23 (21.83)
40–49	12	115.23 (23.31)	6	121.06 (24.37)
	1	102.37 (18.35)	7	134.59 (18.81)
	2	77.71 (23.25)	8	72.85 (13.31)
	3	79.36 (30.54)	9	66.56 (10.32)
	4	64.61 (18.91)	10	90.11 (14.91)
	5	90.79 (19.42)	11	133.02 (18.82)
50–59	12	106.22 (20.85)	6	113.91 (25.8)
	1	90.42 (22.98)	7	125.72 (16.61)
	2	68.82 (22.45)	8	69.71 (13.31)
	3	75.60 (27.31)	9	64.15 (10.04)
	4	57.25 (17.47)	10	84.34 (14.71)
	5	79.10 (19.03)	11	121.29 (20.38)
60–69	12	108.75 (27.82)	6	98.07 (22.55)
	1	87.30 (23.82)	7	115.78 (23.61)
	2	61.34 (20.80)	8	65.82 (14.46)
	3	58.32 (17.31)	9	60.01 (10.57)
	4	52.65 (14.14)	10	73.58 (19.36)
	5	72.52 (19.06)	11	106.68 (29.18)

**Table 4:** Age-related changes in RNFL thickness: Pearson’s correlation coefficient.

Quadrant Sector	Correlation Coefficient with Age (*p*-Value)	Clock Hour Sector	Correlation Coefficient with Age (*p*-Value)
Average	−0.27 (**<0.001**)	12 (Superior)	−0.08 (0.195)
Superior	−0.23 (**<0.001**)	1 (Superior-Nasal)	−0.22 (**<0.001**)
Nasal	−0.12 (0.061)	2 (Nasal-Superior)	−0.14 (**0.02**)
Inferior	−0.26 (**<0.001**)	3 (Nasal)	−0.07 (0.29)
temporal	−0.20 (**0.001**)	4 (Nasal-Inferior)	−0.09 (0.154)
		5 (Inferior-Nasal)	−0.23 (**<0.001**)
		6 (Inferior)	−0.20 (**0.001**)
		7 (Inferior-Temporal)	−0.24 (**<0.001**)
		8 (Temporal-Inferior)	−0.16 (**0.011**)
		9 (Temporal)	−0.14 (**0.022**)
		10 (Temporal-Superior)	−0.20 (**0.001**)
		11 (Superior-Temporal)	−0.25 (**<0.001**)

Bold *p* values indicate statistical significance (*p* < 0.05). RNFL: retinal nerve fiber layer. Age-related changes in RNFL thickness: Pearson’s correlation coefficient.

**Table 5:** Age-stratified macular thickness by sector.

Sector		Macular Thickness (μm), Mean (SD)					
		All	20–29	30–39	40–49	50–59	60–69
Center		243.70 (19.25)	245.21 (17.89)	241.61 (12.48)	244.99 (19.63)	247.62 (22.07)	235.01 (22.12)
Inner ring	Superior	318.39 (14.72)	316.10 (14.70)	317.31 (14.25)	321.69 (15.58)	321.47 (13.73)	310.59 (11.66)
	Nasal	321.76 (15.03)	319.72 (15.34)	321.40 (12.85)	324.40 (16.11)	324.55 (14.52)	314.45 (13.84)
	Inferior	318.02 (13.86)	315.73 (13.46)	319.46 (14.64)	319.32 (14.17)	319.97 (13.27)	312.86 (12.47)
	Temporal	305.12 (14.11)	302.05 (14.21)	303.94 (13.56)	307.30 (15.30)	308.64 (13.27)	300.61 (11.34)
Outer ring	Superior	277.09 (13.05)	273.83 (11.50)	277.82 (14.62)	280.29 (14.42)	277.45 (10.50)	272.73 (11.44)
	Nasal	297.01 (14.58)	294.40 (12.26)	297.52 (15.29)	300.67 (15.01)	298.09 (13.11)	289.69 (15.47)
	Inferior	263.90 (13.38)	260.48 (11.46)	264.56 (16.28)	266.06 (13.57)	264.74 (12.48)	261.54 (11.26)
	Temporal	253.34 (12.17)	249.64 (11.92)	254.97 (13.75)	255.10 (12.66)	254.71 (10.87)	249.95 (9.08)

**Table 6:** Age-stratified macular thickness by retinal layer.

Layer	Sector	All	20–29	30–39	40–49	50–59	60–69
GCC (μm), mean (SD)	Average	111.70 (7.47)	110.11 (6.94)	112.86 (7.29)	113.42 (8.14)	111.19 (7.11)	109.06 (6.51)
	Superior	115.76 (8.47)	114.29 (8.31)	116.26 (8.15)	118.24 (9.07)	115.13 (7.61)	112.35 (7.71)
	S_N	118.61 (8.46)	118.27 (8.27)	119.55 (7.54)	120.41 (9.05)	117.99 (8.14)	114.41 (8.06)
	N_I	118.21 (8.20)	117.52 (7.26)	119.94 (7.82)	119.80 (8.98)	117.49 (7.76)	113.92 (7.53)
	Inferior	114.08 (8.26)	112.27 (6.63)	116.17 (8.30)	115.28 (9.54)	113.27 (7.51)	112.04 (7.61)
	I_T	103.02 (7.75)	100.33 (6.85)	104.34 (7.83)	104.19 (8.31)	102.84 (8.04)	102.56 (6.24)
	T_S	100.52 (7.62)	97.98 (7.21)	100.87 (7.16)	102.58 (8.25)	100.44 (7.95)	99.08 (5.48)
IPL (μm), mean (SD)	Average	111.79 (7.49)	110.18 (6.94)	112.95 (7.32)	113.52 (8.15)	112.28 (7.11)	109.17 (6.52)
	Superior	111.62 (7.74)	110.17 (7.59)	112.23 (7.29)	113.73 (8.25)	111.18 (7.41)	108.63 (6.81)
	Inferior	111.76 (7.62)	110.02 (6.58)	113.47 (7.59)	113.08 (8.54)	111.19 (7.21)	109.51 (6.66)
RPE (μm), mean (SD)	Average	281.77 (11.91)	278.84 (11.15)	282.30 (13.22)	284.34 (12.32)	283.20 (10.66)	276.87 (9.96)
	Fovea	196.32 (17.30)	199.37 (16.75)	189.76 (13.16)	196.41 (16.28)	201.43 (16.89)	193.27 (23.17)
	Center	243.70 (19.25)	245.21 (17.89)	241.61 (12.48)	244.99 (19.63)	247.62 (22.07)	235.01 (22.12)
	Superior	283.83 (12.32)	281.02 (11.62)	283.86 (12.91)	287.00 (13.23)	285.30 (10.61)	278.10 (10.47)
	Inferior	279.70 (12.80)	276.68 (11.03)	280.70 (13.83)	281.67 (12.31)	281.15 (11.26)	275.61 (10.09)

**Table 7:** Comparison of population distribution: Huvitz and Cirrus OCT.

		Huvitz OCT Normative Date	Cirrus OCT Normative Data 1	Cirrus OCT Normative Data 2	*p*-Value ^1^	*p*-Value ^2^
Sample Size, Eyes, N		262	295	302		
Age, mean (SD), range		43.65 (12.89), 20–69	43.86 (10.33), 20–65	42.90 (16.15), 20–79	0.833	0.533
Population distribution, N (%)	20s	49 (18.70%)	27 (9.15%)	69 (22.85%)	**<0.001**	**0.081**
	30s	51 (19.47%)	83 (28.14%)	62 (20.53%)		
	40s	76 (29.01%)	90 (30.51%)	62 (20.53%)		
	50s	54 (20.61%)	75 (25.42%)	56 (18.54%)		
	60s	32 (12.21%)	20 ^†^ (6.78%)	53 ^‡^ (17.55%)		
Sex (Male) N, (%)		135 (51.53%)	160 (54.24%)	-	0.579	-
Laterality (OD) N, (%)		133 (50.76%)	147 (49.83%)	-	0.893	-
**Quadrants**		**RNFL thicknesses (μm)**			**Adjusted** ***p*-value ^1^**	**Adjusted** ***p*-value ^2^**
		**Huvitz** **(*N* = 262)**	**Cirrus 1** **(*N* = 295)**	**Cirrus 2** **(*N* = 302)**		
Mean average, mean (SD)		91.13 (13.00)	98.26 (9.27)	95.08 (3.47)	**<0.001**	**<0.001**
Superior, mean (SD)		111.85 (18.53)	124.71 (16.68)	121.80 (5.71)	**<0.001**	**<0.001**
Nasal, mean (SD)		68.35 (20.03)	69.24 (10.68)	69.43 (1.77)	0.521	0.385
Inferior, mean (SD)		110.40 (19.90)	128.45 (17.26)	122.41 (4.79)	**<0.001**	**<0.001**
Temporal, mean (SD)		73.93 (10.89)	70.52 (12.50)	66.58 (2.29)	**<0.001**	**<0.001**

Bold *p* values indicate statistical significance (*p* < 0.05). SD: standard deviation. The *p*-value ^1^ represents the difference between Huvitz OCT data and Cirrus OCT data 1, while *p*-value ^2^ indicates the comparison of the differences between Huvitz OCT data and Cirrus OCT data 2. ^†^ 60–65, ^‡^ 60–79.

## Data Availability

The datasets generated and analyzed in the current study are available from the corresponding author upon reasonable request.

## Supplementary Materials

The following supporting information can be downloaded at https://www.mdpi.com/article/10.3390/jcm14124258/s1: Figure S1: Quadrants and clock-hour sectors (Right eye); Table S1: Post-hoc Tukey’s test results for each quadrant; Table S2: Percentiles of RNFL thickness for the Huvitz HOCT-1F; Table S3: Percentiles of RNFL thickness for the Huvitz HOCT-1F by quadrant; Table S4: Percentiles of RNFL thickness for the Huvitz HOCT-1F by clock-hour direction; Table S5: Percentile range analysis of GCC, IPL, and RPE thickness; Table S6: Age-stratified percentile range analysis of GCC thickness in the S_N and N_I sectors; Table S7: Age-related changes in macular parameters, Pearson’s correlation coefficient; Table S8: Age-stratified normative data comparison with Cirrus OCT by quadrant.
