# Supplementary material for: Establishment of Normative Retinal Nerve Fiber Layer Thickness in Healthy Koreans Using Huvitz Optical Coherence Tomography and Comparison with Cirrus OCT
Source: J Clin Med. 2025 Jun 15;14(12):4258. doi: 10.3390/jcm14124258 (PMC12194314; doi:10.3390/jcm14124258)
Supplement: Supplementary file 1 [file jcm-14-04258-s001.zip › jcm-3690730-supplementary.pdf]

Supplementary Information

Establishment of Normative Retinal Nerve Fiber Layer Thickness in Healthy Koreans Using Huvitz Optical Coherence Tomography and Comparison with Cirrus OCT

Heesuk Kim [1], Ji Eun Park [2] and Wungrak Choi [1,*]

[1] Institute of Vision Research, Department of Ophthalmology, Yonsei University College of Medicine, Seoul 03722, Republic of Korea; kimhseye@yuhs.ac
[2] Yonsei University College of Medicine, Seoul 03722, Republic of Korea; jieun.park19@med.yuhs.ac
* Correspondence: wungrakchoi@yuhs.ac; Tel.: +82-2-2019-3440

Figure S1. Quadrants and clock-hour sectors (Right eye)

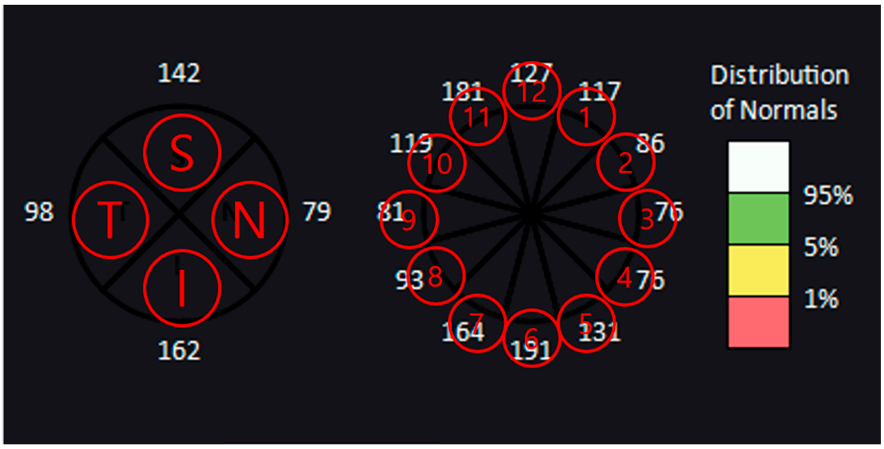

Table S1. Post-hoc Tukey's test results for each quadrant

Age groups	Average	Superior	Nasal	Inferior	Temporal
20–29 vs. 30–39	0.993	1	0.999	0.766	0.998
20–29 vs. 40–49	0.561	0.966	0.604	0.747	0.865
20–29 vs. 50–59	0.376	0.095	0.995	0.657	0.893
20–29 vs. 60–69	**<0.001**	**0.006**	0.085	**0.003**	**0.007**
30–39 vs. 40–49	0.832	0.960	0.412	1	0.975
30–39 vs. 50–59	0.162	**0.092**	1	0.078	0.703
30–39 vs. 60–69	**<0.001**	**0.006**	0.137	**<0.001**	**0.002**
40–49 vs. 50–59	**<0.001**	**0.006**	0.312	0.050	0.262
40–49 vs. 60–69	**<0.001**	**<0.001**	**0.001***	**<0.001**	**<0.001**
50–59 vs. 60–69	**0.028**	0.705	0.168	0.082	0.062

Bold p values indicate statistical significance ($p < 0.05$).

Table S2. Percentiles of RNFL thickness for the Huvitz HOCT-1F

Age group	RNFL thickness (μm)			
	**<1%**	**1–5%**	**5–95%**	**95–100%**
All	<60.89	60.89–69.75	69.75–112.51	>112.51
20–29	<72.17	72.17–79.15	79.15–112.83	>112.83
30–39	<57.78	57.78–68.35	68.35–119.35	>119.35
40–49	<66.55	66.55–73.61	73.61–107.67	>107.67
50–59	<62.91	62.91–70.34	70.34–106.18	>106.18
60–69	<52	52.00–61.77	61.77–108.90	>108.90

The averages for each quadrant were used to determine the range.

Table S3. Percentiles of RNFL thickness for the Huvitz HOCT-1F by quadrant

Age group	Quadrants	RNFL thickness (µm)			
		<1 %	1–5%	5–95%	95–100%
All	Superior	<88.45	88.45–94.87	94.87–125.85	>125.85
	Nasal	<42.08	42.08–49.54	49.54–85.54	>85.54
	Inferior	<79.15	79.15–87.77	87.77–129.39	>129.39
	Temporal	<48.36	48.36–55.63	55.63–90.68	>90.68
20–29	Superior	<82.26	82.26–92.67	92.67–142.89	>142.89
	Nasal	<23.59	23.59–37.65	37.65–105.48	>105.48
	Inferior	<77.46	77.46–89.23	89.23–146.03	>146.03
	Temporal	<51.08	51.08–58.68	58.68–95.34	>95.34
30–39	Superior	<62.51	62.51–77.94	77.94–152.39	>152.39
	Nasal	<20.95	20.95–35.37	35.37–104.93	>104.93
	Inferior	<55.23	55.23–72.59	72.59–156.3	>156.3
	Temporal	<54.47	54.47–60.68	60.68–90.62	>90.62
40–49	Superior	<80.53	80.53–89.54	89.54–132.98	>132.98
	Nasal	<19.51	19.51–33.73	33.73–102.32	>102.32
	Inferior	<71.68	71.68–82.81	82.81–136.53	>136.53
	Temporal	<49.63	49.63–56.66	56.66–90.57	>90.57
50–59	Superior	<71.3	71.3–82.16	82.16–134.56	>134.56
	Nasal	<25.37	25.37–37.41	37.41–95.5	>95.5
	Inferior	<66.44	66.44–78.07	78.07–134.2	>134.2
	Temporal	<48.29	48.29–55.27	55.27–88.95	>88.95
60–69	Superior	<48.84	48.84–65.24	65.24–144.34	>144.34
	Nasal	<30.55	30.55–40.51	40.51–88.53	>88.53
	Inferior	<60.54	60.54–72.63	72.63–130.94	>130.94
	Temporal	<41.18	41.18–49.7	49.7–90.81	>90.81

The averages for each quadrant were used to determine the range.

Table S4. Percentiles of RNFL thickness for the Huvitz HOCT-1F by clock-hour direction

Age group	Clock-hour sector	RNFL thickness (µm)			
		<1%	1–5%	5–95%	95–100%
All	12	<75.49	75.49–85.99	85.99–136.64	>136.64
	1	<58.03	58.03–69.21	69.21–123.19	>123.19
	2	<32.45	32.45–43.75	43.75–98.29	>98.29
	3	<49.91	49.91–56.54	56.54–88.52	>88.52
	4	<22.36	22.36–33.12	33.12–85.02	>85.02
	5	<55.79	55.79–763.97	63.97–103.43	>103.43
	6	<70.75	780.75–839.43	83.43–144.59	>144.59
	7	<94.2	94.102–104.12	104.12–151.96	>151.96
	8	<33.03	33.03–44.1	44.1–97.46	>97.46
	9	<51.95	51.95–55.56	55.56–72.94	>72.94
	10	<43.86	43.86–55.74	55.74–113.1	>113.1
	11	<82.77	82.77–94.73	94.73–152.41	>152.41
20–29	12	<61.63	61.63–77.21	77.21–152.41	>152.41
	1	<50.82	50.82–66.75	66.75–143.59	>143.59
	2	<25.14	25.14–40.31	40.31–113.47	>113.47
	3	<9.54	9.54–28.95	28.95–122.6	>122.6
	4	<13.23	13.23–27.53	27.53–96.48	>96.48
	5	<45.07	45.07–59.02	59.02–126.33	>126.33
	6	<65.54	65.54–82.48	82.48–164.17	>164.17
	7	<88.16	88.16–102.44	102.44–171.35	>171.35
	8	<47.57	47.57–55.42	55.42–93.32	>93.32
	9	<43.45	43.45–50.27	50.27–83.17	>83.17
	10	<50.82	50.82–62.29	62.29–117.59	>117.59
	11	<88.33	88.33–101.53	101.53–165.19	>165.19
30–39	12	<41.48	41.48–62.61	62.61–164.55	>164.55
	1	<35.32	35.32–54.86	54.86–149.16	>149.16
	2	<14	14–31.83	31.83–117.85	>117.85
	3	<5.96	5.96–26.09	26.09–123.18	>123.18
	4	<12.87	12.87–26.97	26.97–94.97	>94.97
	5	<20.46	20.46–40.7	40.7–138.34	>138.34
	6	<40.59	40.59–63.87	63.87–176.22	>176.22
	7	<77.29	77.29–93.84	93.84–173.72	>173.72
	8	<42.89	42.89–51.75	51.75–94.47	>94.47
	9	<46.05	46.05–51.85	51.85–79.85	>79.85
	10	<59.31	59.31–67.72	67.72–108.27	>108.27
	11	<79.11	79.11–94	94–165.83	>165.83
40–49	12	<57.5	57.5–73.4	73.4–150.07	>150.07
	1	<54.59	54.59–67.1	67.1–127.46	>127.46
	2	<17.62	17.62–33.48	33.48–109.98	>109.98
	3	<1.84	1.84–22.68	22.68–123.17	>123.17
	4	<15.41	15.41–28.31	28.31–90.53	>90.53
	5	<39.59	39.59–52.83	52.83–116.73	>116.73
	6	<58.44	58.44–75.05	75.05–155.22	>155.22
	7	<85.34	85.34–98.17	98.17–160.04	>160.04
	8	<40.25	40.25–49.32	49.32–93.1	>93.1
	9	<40.53	40.53–47.57	47.57–81.52	>81.52
	10	<50.39	50.39–60.56	60.56–109.61	>109.61
	11	<80.96	80.96–93.79	93.79–155.71	>155.71
50–59	12	<61.86	61.86–76.08	76.08–144.66	>144.66
	1	<40.3	40.3–55.98	55.98–131.58	>131.58
	2	<17.19	17.19–32.5	32.5–106.35	>106.35
	3	<8.11	8.11–26.74	26.74–116.57	>116.57
	4	<17.63	17.63–29.54	29.54–87.01	>87.01
	5	<36.98	36.98–49.97	49.97–112.59	>112.59
	6	<51.47	51.47–69.06	69.06–153.93	>153.93

	7	<87	87–98.32	98.32–152.96	>152.96
	8	<38.83	38.83–47.91	47.91–91.71	>91.71
	9	<40.23	40.23–47.07	47.07–80.09	>80.09
	10	<48.71	48.71–58.74	58.74–107.13	>107.13
	11	<73.5	73.5–87.4	87.4–154.46	>154.46
60–69	12	<43.94	43.94–62.92	62.92–154.46	>154.46
	1	<34.04	34.04–50.28	50.28–128.65	>128.65
	2	<18.19	18.19–32.37	32.37–100.81	>100.81
	3	<29.82	29.82–41.62	41.62–98.57	>98.57
	4	<23.96	23.96–33.6	33.6–80.13	>80.13
	5	<32.61	32.61–45.61	45.61–108.31	>108.31
	6	<54.55	54.55–69.93	69.93–144.12	>144.12
	7	<66.45	66.45–82.55	82.55–160.22	>160.22
	8	<34.43	34.43–44.3	44.3–91.87	>91.87
	9	<37.79	37.79–45	45–79.79	>79.79
	10	<35.22	35.22–48.43	48.43–112.14	>112.14
	11	<48.33	48.33–68.23	68.23–164.22	>164.22

Table S5. Percentile range analysis of GCC, IPL, and RPE thickness

Layer (μm)	Sector	<1%	1–5%	5–95%	95–100%
GCC	Average	<94.3	94.3–99.4	99.4–123.99	>123.99
	Superior	<96.06	96.06–101.84	101.84–129.69	>129.69
	S_N	<98.92	98.92–104.69	104.69–132.52	>132.52
	N_I	<99.12	99.12–104.71	104.71–131.71	>131.71
	Inferior	<94.85	94.85–100.49	100.49–127.67	>127.67
	I_T	<84.98	84.98–90.27	90.27–115.77	>115.77
	T_S	<82.8	82.8–87.99	87.99–113.05	>113.05
IPL	Average	<93.5	93.5–98.91	98.91–125.01	>125.01
	Superior	<95.56	95.56–100.32	100.32–123.26	>123.26
	Inferior	<91.06	91.06–97.18	97.18–126.71	>126.71
RPE	Average	<265.52	265.52–269.73	269.73–293.1	>293.1
	Fovea	<139.8	139.8–154.88	154.88–238.67	>238.67
	Center	<189.84	189.84–203.7	203.7–280.71	>280.71
	Superior	<267.19	267.19–271.48	271.48–295.27	>295.27
	Inferior	<261.12	261.12–265.97	265.97–292.93	>292.93

GCC: ganglion cell complex; IPL: inner plexiform layer; RPE: retinal pigment epithelium; S_N: superior-nasal; N_I: nasal-inferior; I_T: inferior-temporal; T_S: temporal-superior.

Table S6. Age-stratified percentile range analysis of GCC thickness in the S_N and N_I sectors

Age group	Sectors	GCC thickness, µm			
		**<1%**	**1–5%**	**5–95%**	**95–100%**
20–29	S_N	<100.78	100.78–106.48	106.48–133.95	>133.95
	N_I	<102.98	102.98–107.97	107.97–132.03	>132.03
30–39	S_N	<101.96	101.96–107.1	107.1–131.91	>131.91
	N_I	<101.02	101.02–106.35	106.35–132.08	>132.08
40–49	S_N	<97.38	97.38–103.55	103.55–133.34	>133.34
	N_I	<97.13	97.13–103.25	103.25–132.81	>132.81
50–59	S_N	<98.71	98.71–104.26	104.26–131.05	>131.05
	N_I	<99.11	99.11–104.4	104.4–129.91	>129.91
60–69	S_N	<98.07	98.07–103.53	103.53–129.88	>129.88
	N_I	<100.47	100.47–105.6	105.6–130.38	>130.38

GCC: ganglion cell complex; S_N: superior-nasal; N_I: nasal-inferior.

Table S7. Age-related changes in macular parameters: Pearson's correlation coefficient

	Correlation coefficient with age (p-value)		Correlation coefficient with age (p-value)
**Macular (ETDRS)**		**GCC**	
Center	-0.07 (0.233)	Average	-0.08 (0.213)
Inner ring		Superior	-0.07 (0.261)
Superior	-0.03 (0.587)	S_N	-0.14 (**0.027**)
Nasal	-0.04 (0.476)	N_I	-0.16 (**0.011**)
Inferior	-0.05 (0.414)	Inferior	-0.08 (0.189)
Temporal	0.02 (0.731)	I_T	0.02 (0.783)
Outer ring		T_S	0.02 (0.807)
Superior	-0.03 (0.632)	**IPL**	
Nasal	-0.06 (0.298)	Average	-0.08 (0.221)
Inferior	0.02 (0.787)	Superior	-0.07 (0.260)
Temporal	0.00 (0.978)	Inferior	-0.08 (0.201)
		**RPE**	
		Average	-0.03 (0.647)
		Fovea	0.02 (0.689)
		Center	-0.07 (0.233)
		Superior	-0.04 (0.568)
		Inferior	-0.02 (0.75)

Bold p values indicate statistical significance ($p < 0.05$).

Table S8. Age-stratified normotive data comparison with Cirrus OCT by quadrant

Quadrant	Age group	Huvitz vs. Cirrus 1 (Adjusted p-value)	Huvitz vs. Cirrus 2 (Adjusted p-value)
Superior	20–29	**< 0.001**	**< 0.001**
	30–39	**< 0.001**	**< 0.001**
	40–49	**< 0.001**	**< 0.001**
	50–59	**< 0.001**	**< 0.001**
	≥ 60	**< 0.001**	**< 0.001**
Nasal	20–29	0.456	0.612
	30–39	0.811	0.742
	40–49	0.385	0.529
	50–59	0.295	0.312
	≥ 60	0.180	0.240
Inferior	20–29	**< 0.001**	**< 0.001**
	30–39	**< 0.001**	**< 0.001**
	40–49	**< 0.001**	**< 0.001**
	50–59	**< 0.001**	**< 0.001**
	≥ 60	**< 0.001**	**< 0.001**
Temporal	20–29	0.652	0.324
	30–39	0.586	0.412
	40–49	**0.042**	**0.018**
	50–59	**0.009**	**0.007**
	≥ 60	**0.004**	**0.002**

Bold p values indicate statistical significance ($p > 0.05$).
